# Lethal infection caused by *Tetratrichomonas gallinarum* in black swans (*Cygnus atratus*)

**DOI:** 10.1186/s12917-021-02894-x

**Published:** 2021-05-13

**Authors:** Shengyong Feng, Han Chang, Yutian Wang, Fubing Luo, Qiaoxing Wu, Shuyi Han, Hongxuan He

**Affiliations:** 1 National Research Center for Wildlife Borne Diseases, Institute of Zoology, Chinese Academy of Sciences, 1-5 Beichenxilu, Chaoyang District, Beijing, 100101 PR China; 2 College of Life Sciences, University of Chinese Academy of Sciences, Chaoyang District, Beijing, 100101 China; 3 Beijing General Station of Animal Husbandry, Chaoyang District, Beijing, 100107 China; 4 Beijing Center for Animal Disease Control, Beijing, China; 5 Shaanxi Institute of zoology, Xi’an, 710032 Shaanxi China

**Keywords:** Black swan, Cecum, China, Liver, *T. gallinarum*

## Abstract

**Background:**

*Tetratrichomonas gallinarum* is parasitic protozoa with a wide host range. However, its lethal infection is rare reported.

**Case presentation:**

Here, we described the first lethal cases of *T. gallinarum* infection in black swans in China. Five black swans died within a week in succession without obvious symptoms except mild diarrhea. At necropsy, severe lesions were observed in caeca with thickened caecal walls and hemorrhages in the mucosa. A large number of moving trophozoites were found in the contents of the cecum by microscopic examination. The livers were enlarged with multiple bleeding spots on the surface. Histopathology of the livers showed mononuclear cell infiltration and moderate hyperplasia of fibrous tissue. The histopathology of the cecum showed that the villi of the cecum were edematous. Finally, the presence of *T. gallinarum* was determined by specific PCR andin-situ hybridization assay. Additionally, common pathogens that can cause similar symptoms were excluded.

**Conclusions:**

The death of the black swan was caused by *T. gallinarum*, suggesting that the parasite might be a new threat to the *Cygnus* birds.

## Background

*Tetratrichomonas gallinarum* is parasitic protozoa with a wide host range [1]. Owing to sick birds are usually co-infected with other pathogens and artificially infected animals rarely develop symptoms, the pathogenicity of *T. gallinarum* is controversial [2–4]. Moreover, lesions caused by *T. gallinarum* in birds were sporadically reported in some countries, such as in chukar partridges, mockingbird, Waldrapp ibis and white pelican from America [5–8], in duck from Germany [9], in red-legged partridges from Great Britain [10], and in Layer chickens from the Netherlands [11]. Here, we described the first fatal case of black swans (*Cygnus atratus*) associated with *T. gallinarum* infection in China, and the threat of the protozoa to *Cygnus* birds must be considered.

## Case presentation

In August 2019, five adult black swans from a wetland park of Beijing died within a week in succession. Before they died, no obvious symptoms were observed except mild diarrhea.

**Fig. 1:**
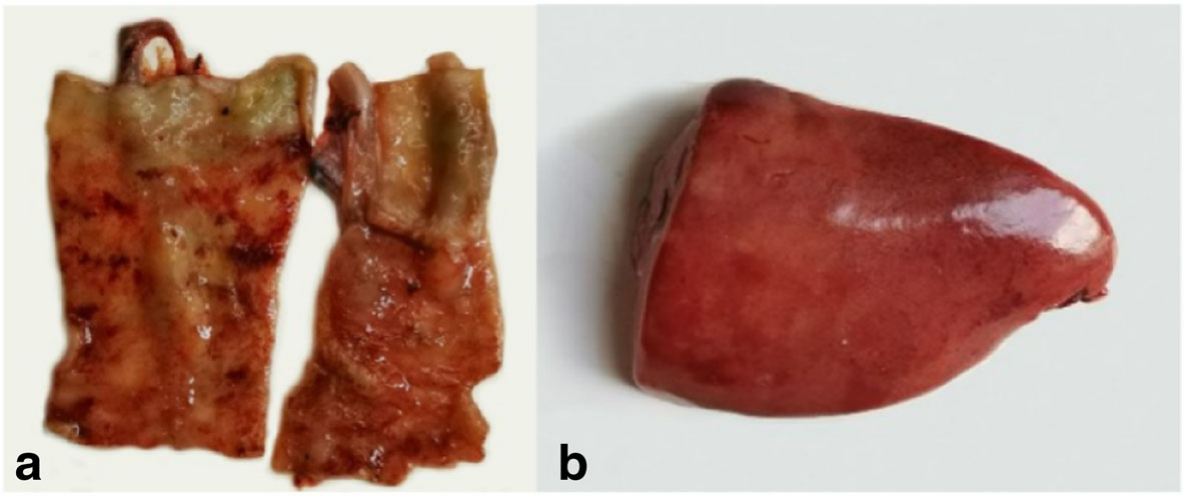
Pathological changes of cecum (**a**) and liver (**b**)

**Fig. 2:**
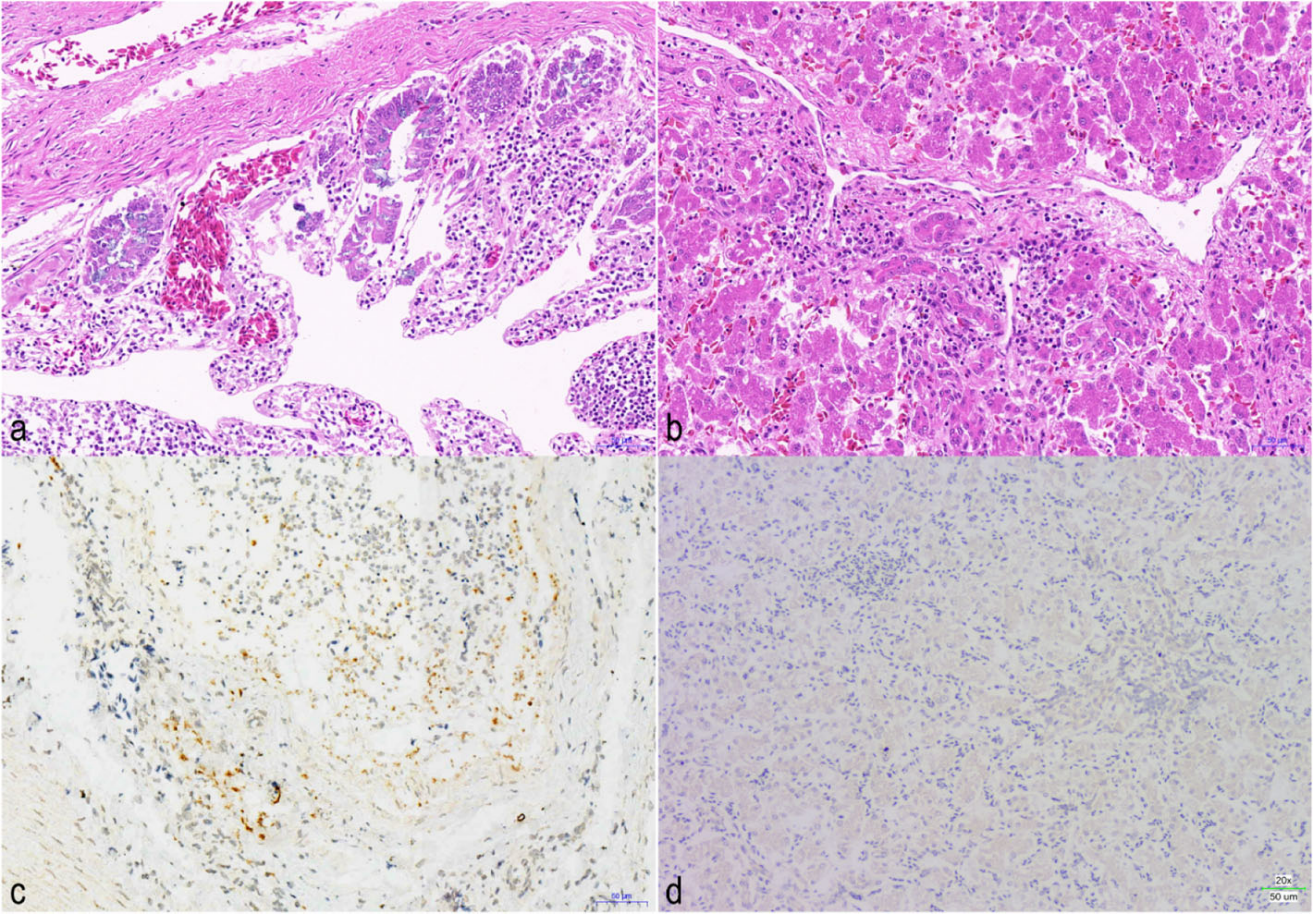
Haematoxylin and eosin staining of the caecum (**a**) and the liver (**b**) of a dead black swans. ISH revealed the presence of *T. gallinarum* in the caecum (**c**) within the localizations as brown-stained cells. The signals of *T. gallinarum* probe in the liver was negative (**d**)

The fresh carcasses were sent to the National Research Center for Wildlife Borne Diseases for postmortem and histopathological examination. At routinely pathological investigation, the ceca were swollen and the mucosa were hemorrhages and anabrosis (Fig. 1a). A large number of moving trophozoites were observed by microscopic examination. The livers were enlarged and accompanied by the color turned dark red and the edge was blunt (Fig. 1b). No visible lesions were found in other organs. Histopathological examination showed that cecal hemorrhage, intestinal villi edema, disordered arrangement, epithelial cells exfoliated, and many parasites were found in lamina propria (Fig. 2a). Vacuolar degeneration of hepatocytes and interlobular bile duct hyperplasia were observed in the liver tissues. A large number of mononuclear inflammatory cells infiltrated between the liver lobules, and the fibrous tissue proliferated moderately (Fig. 2b).

Histological sections from the livers and ceca of the birds were further processed for in situ hybridization (ISH) using the described probe specific for *T. gallinarum* and *H. meleagridis* [12, 13]. The positive signals with the *T. gallinarum* probe were found in the caeca (Fig. 2c) but not in the livers (Fig. 2d). The result of ISH in the caeca and livers showed no signal with the *H. meleagridis* probe.

**Fig. 3:**
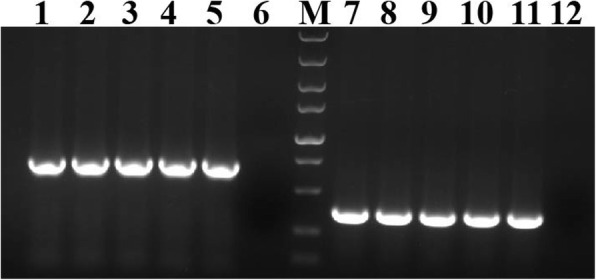
Partial sequence amplification based on 18S rRNA gene and ITS1–5.8S rRNA-ITS2 gene. Lane 1–5, PCR products of18S rRNA gene. Lane 7–11, PCR products of ITS1–5.8S rRNA-ITS2 gene. Lane 6 and 12 were negative control. M, marker

**Fig. 4:**
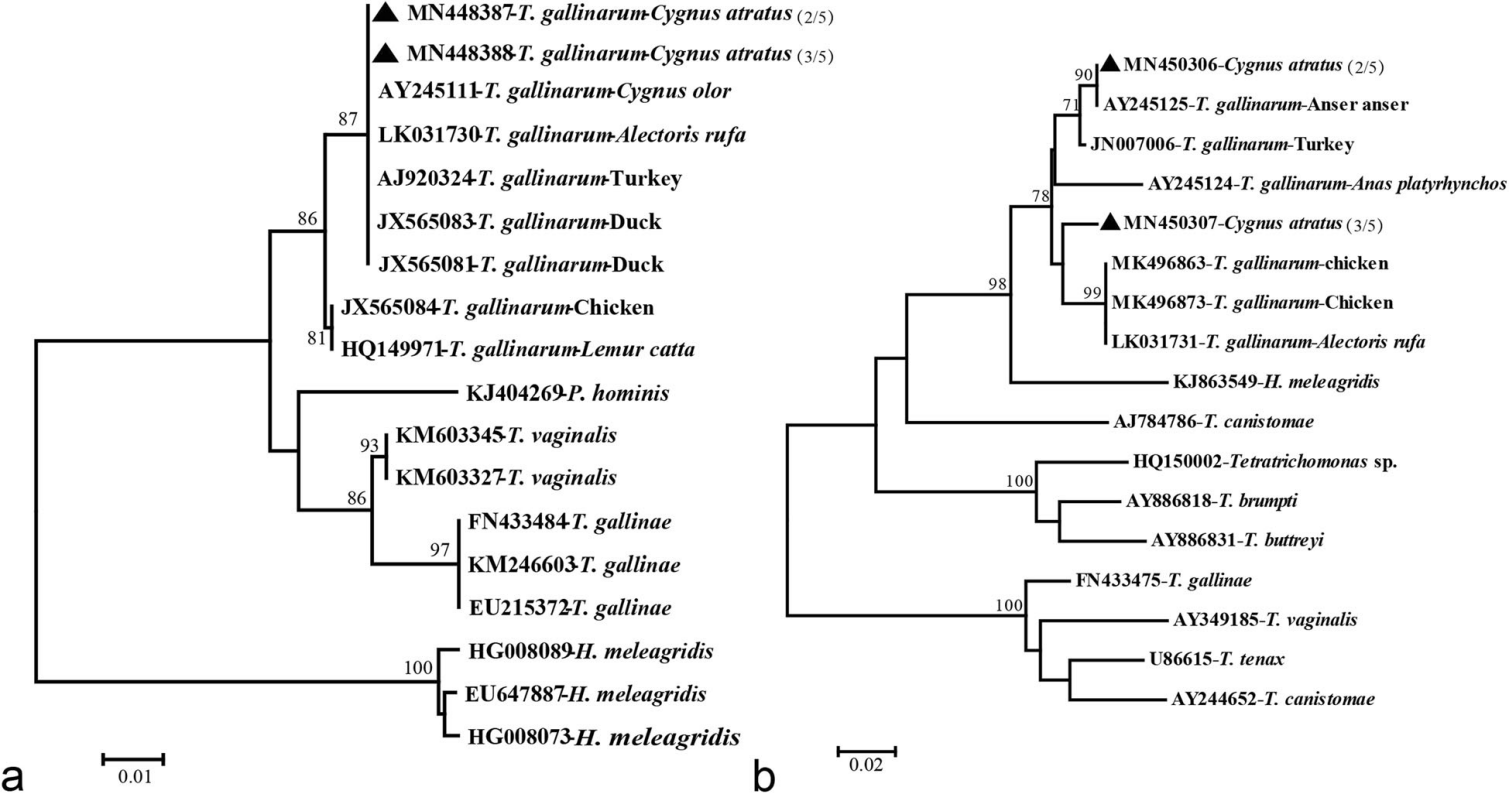
Phylogenetic tree of the trichomonad nucleic acid sequences based on **a** the partial 18S rRNA and **b** ITS1–5.8S rRNA-ITS2 loci. The phylogenetic tree was constructed with a Neighbor-Joining method with the Kimura2-parameter model. Bootstrap values > 70% from 1000 replicates are shown on the nodes. The isolates detected in the current study are shown with solid triangle

Using two trichomonad primer sets, TFR1/R2 and 18S-F/R, the ITS and 18S rRNA region of the isolates were successfully amplified with specific single band size of approximately 350 bp and 600 bp in the gel [14, 15] (Fig. 3), respectively. Notably, the PCR products were subcloned into T-vectors before sequencing to ensure that the specific sequences be successfully sequenced. Both sequences were clustered with the reference sequences of *T. gallinarum* download from GenBank database under phylogenetic analyses (Fig. 4a, b).

Other potential pathogens, such as *Coccidia* spp., *Blastocystis* spp. and hepatitis E virus were negative using the method previously reported [16–18].

Taken together, after eliminating potential pathogens, such as *H. meleagridis*, *Coccidia*, *Blastocystis* spp., hepatitis E virus as well as pathogenic bacteria, the presence of *T. gallinarum* was eventually confirmed by microscopic examination, histopathology, specific PCR amplification and ISH. Therefore, the death of the black swan was likely to be caused by *T. gallinarum.*

## Discussion and conclusions

Though *T. gallinarum* is commonly found gallinaceous and anseriform birds, it seldom causes diseases [19]. The maturity of the immune system may be an important reason for the host to suffer from this parasite, as previous studies have found that most of the dead birds were juveniles or subadults [8, 9]. However, all the dead black swans in the present study were adult, thus the heterogeneity between *T. gallinarum* isolates might also be an important factor result in the differences in pathogenicity among hosts.

Studies conducted by Dimasuay and Rivera shown that *T. gallinarum* can be detected from healthy ducks (*Anas platyrhynchos*) [20], which suggested that the parasite might be commensal in some duck species. In the present study, some healthy ducks shared activity area with the black swans. Thus the *T. gallinarum* recovered from the black swans may be spillover from the ducks.

In conclusion, we described the first fatal case of black swans associated with *T. gallinarum* infection in China, suggesting that the protozoan might be a new threat to the *Cygnus* birds. A comprehensive epidemiological investigation of *T. gallinarum* in *Cygnus* birds is urgently needed in the future.

## Data Availability

The ITS and 18S nucleotide sequences of *T. gallinarum* generated in the present study have been deposited in GenBank database under the accession numbers MN448387and MN448388 as well as MN450306 and MN450307, respectively.

## Abbreviations

ISH: In situ hybridization
ITS: Internal transcribed spacer
